# New Isolated *Metschnikowia pulcherrima* Strains from Apples for Postharvest Biocontrol of *Penicillium expansum* and Patulin Accumulation

**DOI:** 10.3390/toxins13060397

**Published:** 2021-06-02

**Authors:** Laura Settier-Ramírez, Gracia López-Carballo, Pilar Hernández-Muñoz, Angélique Fontana, Caroline Strub, Sabine Schorr-Galindo

**Affiliations:** 1 Packaging Lab., Instituto de Agroquímica y Tecnología de Alimentos, IATA-CSIC, Av. Agustín Escardino 7, 46980 Paterna, Spain; glopez@iata.csic.es (G.L.-C.); phernan@iata.csic.es (P.H.-M.); 2 Qualisud, University Montpellier, Avignon Université, CIRAD, Institut Agro, IRD, Université de La Réunion, 34095 Montpellier, France; angelique.fontana@umontpellier.fr (A.F.); sabine.galindo@umontpellier.fr (S.S.-G.)

**Keywords:** biological control, *Metschnikowia pulcherrima*, *Penicillium expansum*, patulin, epiphytic isolated yeasts, apple

## Abstract

Wild yeasts isolated from the surface of apples were screened for antagonistic activity against *Penicillium expansum*, the main producer of the mycotoxin patulin. Three antagonistic yeasts (Y33, Y29 and Y24) from a total of 90 were found to inhibit *P. expansum* growth. Identification by ITS region sequence and characterization showed that three selected isolates of yeast should be different strains of *Metschnikowia pulcherrima.* Several concentrations of the selected yeasts were used to study their in vitro antifungal effectivity against *P. expansum* on Petri dishes (plates with 63.6 cm^2^ surface) whereas their potential activity on patulin reduction was studied in liquid medium. Finally, the BCA that had the best in vitro antifungal capacity against *P.* and the best patulin degradation capacity was selected to be assessed directly on apples. All the selected strains demonstrated antifungal activity in vitro but the most efficient was the strain Y29. Isolated strains were able to reduce patulin content in liquid medium, Y29 being the only strain that completely reduced patulin levels within 120 h. The application of Y29 as biocontrol agent on the surface of apples inoculated with *P. expansum,* inhibited fungal growth and patulin production during storage. Therefore, the results shown that this yeast strain could be used for the reduction of *P. expansum* and its mycotoxin in apples or apple-based products by adapting the procedure application.

## 1. Introduction

Currently, more than 30% of fruit and vegetables produced worldwide is discarded every year [1]. These losses occur throughout all the supply chain from cultivation to household consumption although most of them happen during the postharvest stage, processing, transportation or storage. The decomposition of fruits by fungal spoilage represents the main cause of those economic losses [2].

*Penicillium expansum* is one of the best-known species of the genus *Penicillium* due to its high relevance as it is a widespread species found in the natural environment both in soil, air and even indoor air [3,4]. Furthermore, *P. expansum* infect a wide range of food products including pome and stone fruits (such as apples, pears, cherries, or peaches) which are the primary targets for this toxigenic fungus. It has also been isolated worldwide from vegetables [5], grains and even in dairy products [6] and sea food [7,8]. This common fungus is responsible for the blue mold decay of fruits (blue mold rot disease also called soft rot), where damaged and bruised fruits soften and brown [9].

Besides the fact that *P. expansum* decreases the quality and increases the economic losses of infected fruits, it can also lead to sanitary consequences since it produces toxins that increase the risk of serious diseases in human consumers [2]. Patulin (4-hydroxy-4H-furo [3,2c]pyran-2[6H]-one) is the main mycotoxin pose by *P. expansum,* but it could also be produced by a wide range of fungi. It is a polyketide-derived mycotoxin product of the secondary metabolism of fungi and it is toxic to humans and animals at very low doses [10]. It can produce acute and chronic toxicity such as genotoxicity, cytotoxicity, mutagenicity and immunotoxicity [11]. Thus, many countries and organizations have established regulatory limits for patulin content. In the European Union, patulin is limited to 50 μg·kg^−1^ in fruit juices, to 25 μg·kg^−1^ in solid fruit products, and to 10 μg·kg^−1^ in products for infants and children [12]. Besides, it is hard to remove patulin from processed products since this mycotoxin is relatively stable to thermal treatments and resistant to acidic conditions.

Synthetic chemical fungicides represent the most common used strategy to contain *P. expansum* at the preharvest and postharvest stages. However, the use of these products can involve potential health risks and fungicide accumulation in agricultural products affecting the safety of the entire food chain. The emergence of new fungal species resistant to fungicides as well as the impact of pesticides on the environment [2] has also to be considered. Moreover, different strategies have been developed for patulin detoxification, being both physical and chemical decontamination the most used. Nevertheless, some problems exist in the use of available methods, like safety issues, probable losses in the nutritional quality, limited efficiency and high costs [13]. For that, consumers are demanding the use of more ecofriendly alternatives. Given the current legislation and public opinion, BCA such as yeast, filamentous fungi, and bacteria that have been shown to have antagonistic effects against *P. expansum*, have gained significant interest as alternatives to conventional methods currently in use. In contrast with the traditional methods discussed above, BCA are able to persist for long periods after treatment and are able to protect fruit from reinfection [14]. In this sense, BCA are often part of integrated pest management, which aims to combine a variety of control methods (such as crop rotation, use of mixed cropping, use of pest-resistant varieties or targeted use of pesticides), which minimize overall economic, health and environmental risks [15]. Among BCA, yeasts are good candidates because they have relevant characteristics in the selection of a BCA such as simple nutritional requirements. They also can grow rapidly on inexpensive substrates in bioreactors, they are able to colonize dry surfaces, survive for long periods of time and they have a low impact on the environment [16]. Moreover, yeasts do not produce allergenic spores or toxins and do not damage fruits as it could occur with fungi [17]. In addition, most of them have the ability to survive on the surface of fruits contrary to bacteria [2]. Antifungal yeasts can be applied at preharvest and postharvest stages. Indeed, yeasts spraying or dipping are some of the best practical and useful methods developed to control postharvest disease of fruits and vegetables [18].

Among yeasts, strains isolated from the epiphytic microbial community of fruits and vegetables are the most promising BCA since they are phenotypically adapted to this niche. The benefit of belonging to the microbial community naturally established in the target food product can provide their colonization and survival on it when applied in the appropriate concentration [19]. Other authors have also demonstrated that a better disease suppression is observed when growth conditions for both antagonist and fungal pathogen are similar [20]. Therefore, the microbial antagonists isolated from the same environment are appropriate for disease control and are the best sources of antagonistic microorganisms [21,22]. Another advantage of using yeasts as BCA is that more of them exhibited the ability to detoxify patulin in anaerobic or aerobic conditions. This potential was first observed in cider fermented by strains of *Saccharomyces* genus, where patulin levels did not exceed 50 μg·kg^−1^ [23]. Different authors have reported that yeast such as *Pichia caribbica* [24], *Saccharomyces cerevisiae* or *Rhodosporidium kratochvilova* [25] were able to degrade patulin to undetectable levels. In some cases, products of patulin biodegradation by yeasts have been identified as (E)- and (Z)-ascladiol and desoxypatulinic acid, products which are much less toxic than patulin for humans [25,26].

The aim of the current study was to search indigenous yeast strains of apple to act as a potential biocontrol agent against *P. expansum* with the ability to reduce patulin content of fruits and that could be applied directly on the surface of apples as an alternative to conventional pesticides. For this purpose, different yeast strains have been isolated from apples and screened for their antifungal activity against *P. expansum*. An identification and characterization of selected yeasts was also conducted. Finally, the antifungal capacity of the selected yeasts was quantified and their ability to biodegrade patulin was also studied. The yeast that presented the best performance in the in vitro studies was then applied on wounded “Golden delicious” apples inoculated with *P. expansum* to study its potential as a BCA.

## 2. Results

### 2.1. Screening for Antagonistic Yeasts

**Figure 1:**
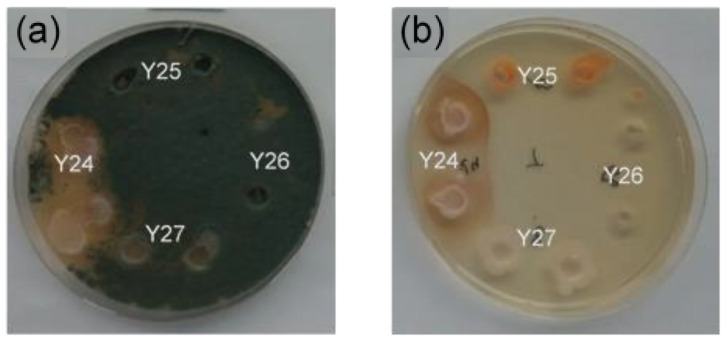
Growth of some isolated yeasts and antifungal activity (**a**) against *P. expansum* after 7 days at 25 °C and growth of those yeasts in PDA Petri dishes (**b**). In the figure, a duplicate of yeasts number 24, 25, 26 and 27 were tested in the same Petri dish.

Ninety yeast strains were isolated from the skin of apples as soon as they were harvested in the orchard. A screening of these isolates for biocontrol efficacy against *P. expansum* growth was carried out in PDA plates previously inoculated with 100 µL of 10^5^ spores/mL of *P. expansum*. As it can be seen in Figure 1, only yeasts that exhibited an inhibition halo were chosen (here in Figure 1a as an example, among yeasts 24, 25, 26 and 27 tested, only yeast 24 exhibited an inhibition halo). The screening showed that only three strains were able to inhibit the growth of the mold. These antifungal yeasts were assigned as Y33, Y29 and Y24.

Yeast strains Y33, Y29 and Y24 were found to be similar in colony characteristics and cell morphologies (results not showed) and the three presented a brown-red halo around wells and around colonies in PDA plates when they grew without *P. expansum* as it can be noticed in Figure 1b for Y24.

### 2.2. Identification and Characterization of Antagonistic Yeast

The identification and characterization of Y33, Y29 and Y24 were carried out. The 5.8S genes were amplified by the PCR technique, which yielded in 390–400 bp for each DNA fragment as it is shown in Figure S1. Sequencing was carried out for the ITS region. The 5.8S rDNA sequences of the isolated yeasts were compared with the homologous sequences contained in the GenBank database by using the BLAST software. The 5.8S rDNA sequence of yeast strains Y33, Y29, and Y24 showed high identity with *Metschnikowia pulcherrima* at 100%, 97.2%, and 100%, respectively. Partial sequences of the 5.8S rRNA gene were deposited at GenBank under the following accession numbers: MW532819 for *M. pulcherrima* strain Y33; MW532956 for *M. pulcherrima* strain Y29 and MW532963 for *M. pulcherrima* strain Y24.

**Figure 2:**
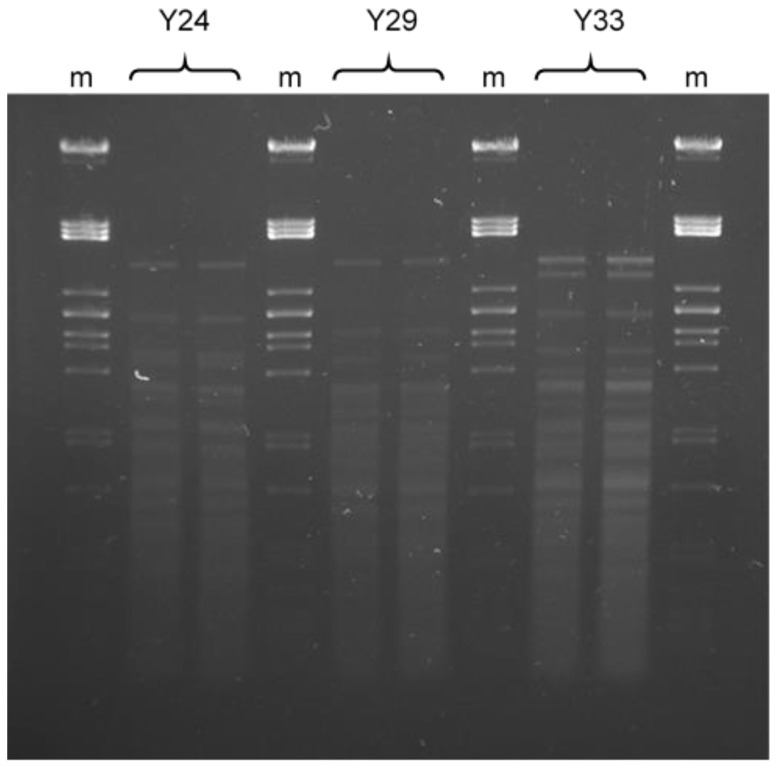
mt DNA patterns obtained after digestion with Hinf I of isolated yeasts. Lanes m correspond to Lambda DNA Pst I ladder.

Additionally, as it is shown in Figure 2, none of the isolated yeasts showed the same mtDNA-RFLP pattern. Therefore, Y33, Y29 and Y24 isolated from apples belong to the *Metschnikowia pulcherrima* species but they are different strains.

### 2.3. Comparison of In Vitro Biocontrol Efficacy of Different Concentrations of Antagonistic Yeasts

**Figure 3:**
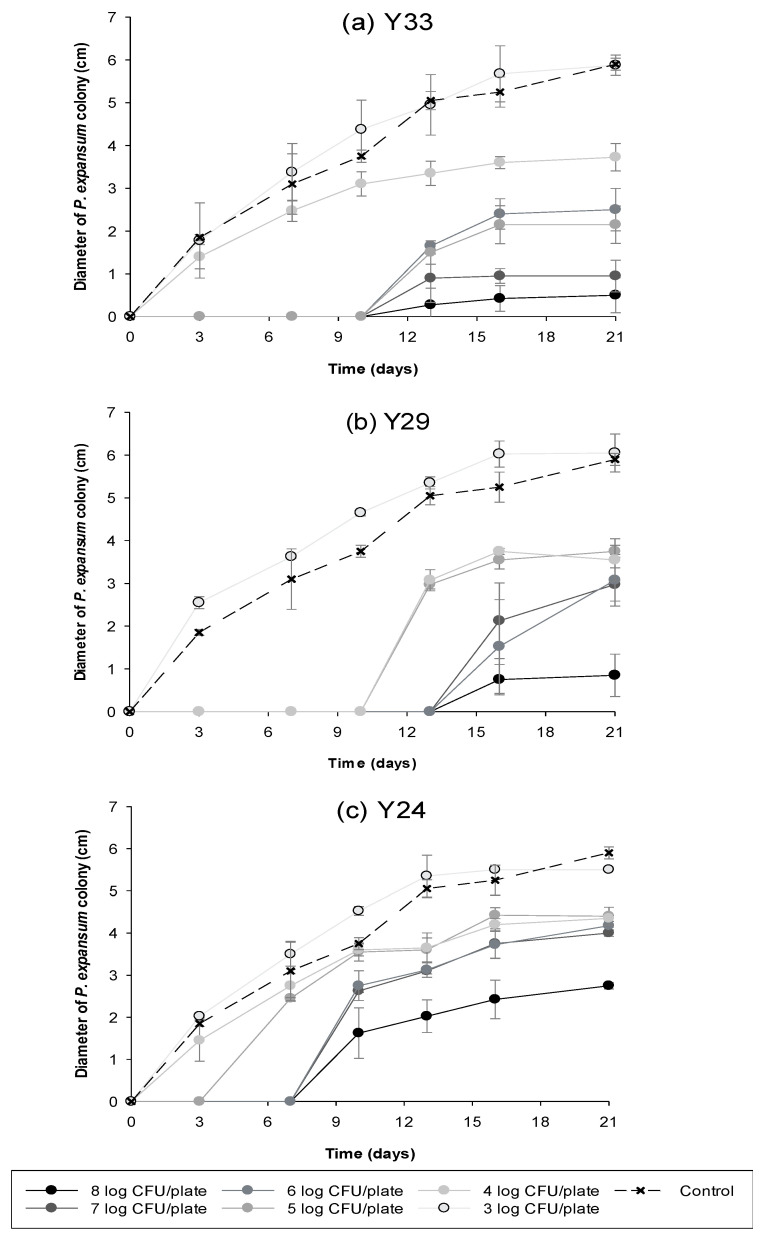
Antifungal activity of different concentrations of (**a**) Y33, (**b**) Y29 and (**c**) Y24 against *Penicillium expansum* (3 log spores/plate) in PDA incubated at 28 °C for 21 days.

Once the strains were isolated and identified, their antifungal activity against *P. expansum* was studied using different yeast concentrations for 21 days at 28 °C, the optimal temperature growth of the pathogen, as can be seen in Figure 3. In all the cases, pathogen incidence was reduced when the antagonistic concentration was increased. The best results were obtained when the concentration of antagonist yeast was greater, 8 log CFU/plate, than *P. expansum* concentration, 3 log spores/plate. The lower antagonist concentration tested, 3 log CFU/plate was not effective against the pathogen.

However, some differences in the antifungal activity were observed among isolated yeasts. The application of the antagonists at the higher concentration significantly inhibited pathogen development obtaining reductions of 91.4%, 85.3% and 52.6% for Y33, Y29 and Y24, respectively after 21 days of incubation at 28 °C. In addition, no *P. expansum* growth was observed until day 13, day 16 and day 10 when Y33, Y29 and Y24 were applied, respectively. Therefore, the results shown that yeast strain Y29 was more effective than the other two strains in reducing the growth of *P. expansum*.

### 2.4. Biodegradation of Patulin by Antagonistic Yeasts in Liquid Medium

#### 2.4.1. Check of Patulin Impact on Yeast Cell Viability

**Figure 4:**
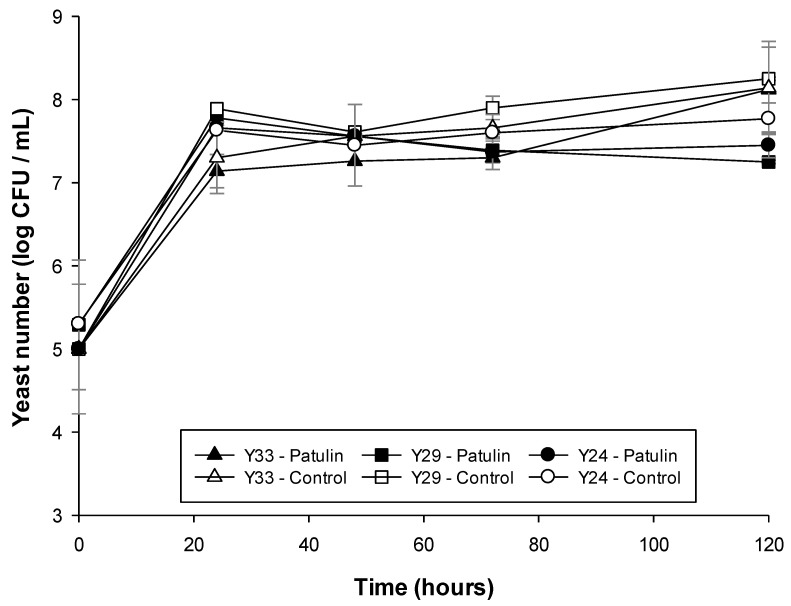
Viability of yeasts strains in contact with patulin in liquid medium at 25 °C for 120 h.

Isolated yeast growth in pepton malt extract broth (PM) medium supplemented with 1000 µg/L patulin is presented in Figure 4. The maximum microbial count for all the yeasts in contact with patulin was observed around 24 h. However, yeasts without patulin were able to increase their growth until the end of the experiment. The presence of patulin slightly restrained the yeast growth and after 120 h, yeast growth in the presence of patulin was less than the control observing reductions between 1.5 and 0.5 log depending on the yeast.

#### 2.4.2. Patulin Reduction by Antagonistic Yeasts

**Figure 5:**
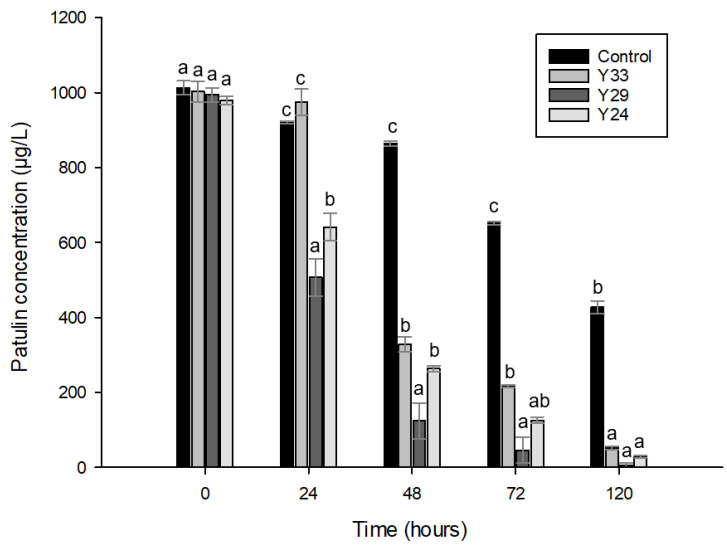
Efficacy of yeast strains on biodegradation of patulin in liquid medium at 25 °C for 120 h. Different letters within a sampling day mean significant differences among samples (*p* < 0.05).

The three isolated strains were evaluated for their potential for patulin degradation under in vitro conditions in PM liquid medium and the results are presented in Figure 5. For the three tested strains, the highest level of patulin degradation was observed for the isolate Y29. In fact, it reduced patulin concentration up to half after 24 h and it was able to completely reduce patulin in 120 h. Y24 had also a great potential to reduce patulin exerting a progressive reduction over time until obtaining a value of 28 µg/L after 120 h. Finally, although Y33 was also able to considerably reduce the patulin, it was the slowest to degrade it. Contrary to the other two strains, after 120 h of incubation, patulin concentration was above the limits established by the EFSA (50 µg/mL). It must be pointed out that no detectable levels of patulin were observed neither in yeast cell walls nor in intracellular metabolites. This indicated that patulin was degraded to another substance and was not absorbed or adsorbed by yeast cells.

### 2.5. Biocontrol Activity of Y29 Applied on Apples

**Figure 6:**
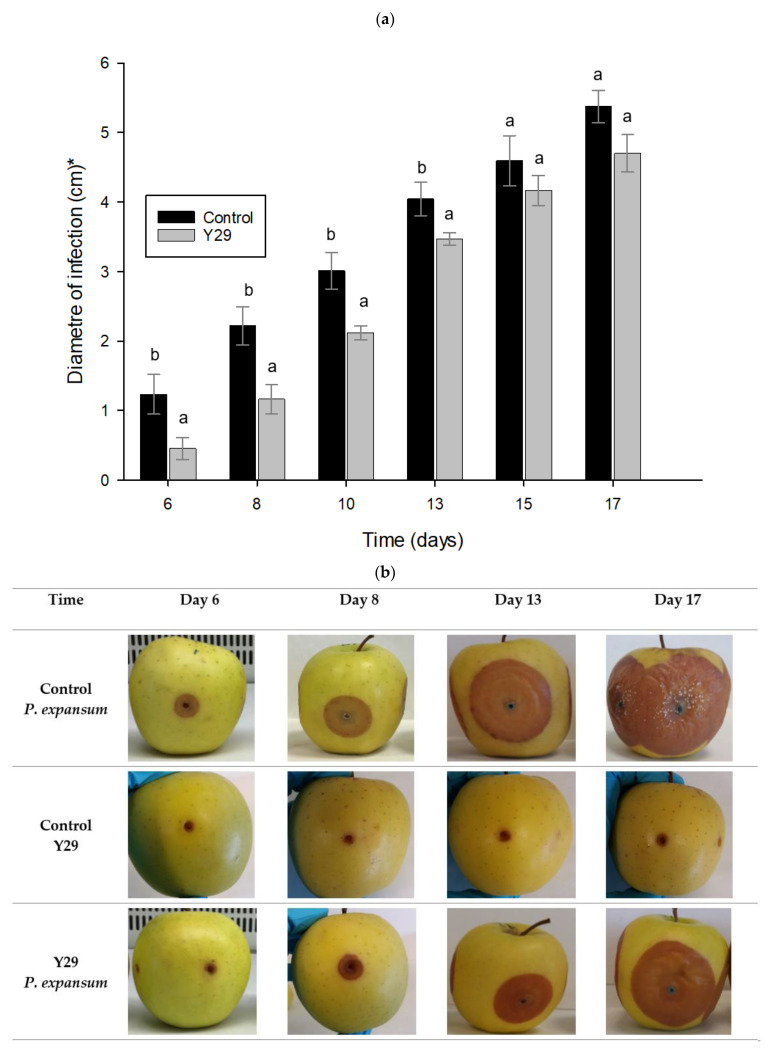
(**a**) Biocontrol activity of Y29 applied on apples artificially inoculated with *Penicillium expansum* stored at 21 °C for 17 days. Different letters within a sampling day mean significant differences among samples (*p* < 0.05). * The diameter of the infection has been subtracted from the diameter of artificially wound made before *P. expansum* inoculation (3 mm diameter) and (**b**) visual effect of the application of Y29 on apples artificially inoculated with *Penicillium expansum* after incubation at 21 °C for 6, 8, 13 and 17 days.

Y29 has shown the best in vitro antifungal capacity against *P. expansum* (Section 2.3) and the best patulin degradation capacity (Section 2.4). Therefore, it was selected to be assessed directly on apples. The initial number of viable yeasts after dipping in 8 log CFU/mL suspension and drying was 6.11 log CFU/apple. The growth of *P. expansum* was monitored by measuring the halo of infection around the 3 mm wound made in the apples and the results are shown Figure 6a.

Pictures of the appearance of control apples and apples treated with BCA and the progression of the infection in 17 days of storage are depicted in Figure 6b. It should be mentioned that the halo size of apples dipped in water (results not shown) and dipped in yeast suspension without *P. expansum* (Figure 6b for Control Y29), did not exceed 2 mm throughout storage time. Thus, neither the oxidative reactions that naturally occur on apple wounds, nor *M. pulcherrima* colonization of the wounds had a significant impact on the development of the halo. When Y29 suspension was applied on apple surface, the disease was delayed during the first ten days of storage, showing a significant reduction in fungal growth; after ten days fungal growth increased rapidly.

### 2.6. Patulin Quantification on Apples

At the end of the storage (after 17 days at 21 °C), apples artificially inoculated with *P. expansum* and subjected to the different treatments were crushed and treated to extract patulin. Patulin was produced in all the samples inoculated with *P. expansum* with a low but significant decrease from 1995.73 ± 103.62 µg/fruit in control apples to 1793.55 ± 86.21 µg/fruit in apples treated with BCA. No patulin was detected in control Y29 (without *P. expansum*) apples. It must be pointed out that each apple was injured with four wounds and each wound was inoculated with a high concentration of *P. expansum*. Therefore, such high amounts of patulin per apple after 17 days of storage period at 21 °C were expected. The amount of patulin is related to the diameter of wound infection in treated apples (as seen in previous section), with highest patulin content corresponding to highest infection.

## 3. Discussion

*Penicillium expansum* is the agent of the blue mold decay considered as the most severe postharvest fungal disease since it causes heavy losses in the transportation and storage and it produces the harmful mycotoxin patulin especially in apple fruit [27]. The use of biocontrol agents could inhibit postharvest fruit pathogens by simple antagonistic interaction. Yeasts such as *Pichia carribbica*, *Candida sake* or *Rhodosporidium paludigenum* among others have been successfully used for controlling fruit diseases [2]. However, the most promising BCA are yeasts isolated from the epiphytic microbial community of fruits as they are phenotypically adapted to this niche. The benefit of belonging to the microbial community naturally established in the target fruit is that it can provide their colonization and survival on it when applied in the appropriate concentration [19]. Besides, to date, the resident microbial load of apples has been found to be considered as the most effective antagonists against pome fruit decay [28].

In the current research new yeast strains isolated from the surface of apples, named Y33, Y29 and Y24, were selected for their efficacy against *P. expansum*. Yeasts were identified as three strains of *Metschnikowia pulcherrima* through sequencing of the ITS region mt-DNA could clearly distinguish that three different strains of *M. pulcherrima* were involved.

In fact, *Metschnikowia* spp. are terrestrial, free-living, often associated with flowers and transmitted to new niches by insects [29]. *M. pulcherrima*, in particular, is considered as one of the 13 resident species on cider apple trees [30]. According to other studies, it has been isolated from apples cultivars from floral parts, from buds and even in wounded apple tissue [28,31].

In vitro experiments revealed that the three strains were effective against *P. expansum* but despite belonged to the same species, they exhibited different antifungal activity. The best results were obtained with Y29. These results are in agreement with previous studies, where different strains of the same yeast species showed different biocontrol abilities because of their genetic background [31,32] as it is often the case in biocontrol interaction. A wider brown-red pigmented inhibition zone of *P. expansum* was observed for Y33, Y29 and Y 24 (Figure 1a). In fact, the main antifungal mechanism of *M. pulcherrima* is the production of a red pigment, pulcherrimin, which accumulates in cells and it is also secreted around the colonies [33]. This pigment forms a chelate complex and immobilizes the iron ions in the growth medium which are essential for the fungal growth and pathogenesis [34]. In addition to the nutritional competition activity, other authors have reported that *M. pulcherrima* can also exert competition by parasitism due to the production of lytic enzymes such as chitinase and volatile antifungal compounds that contribute to the overall antagonistic effects [35,36]. Moreover, for all the isolated yeasts, the higher concentration of yeast produced the more antifungal activity. Other authors have also observed that biocontrol activity of the microbial antagonist was concentration dependent [37]. As it occurs in this experiment, the initial concentration of the antagonist remains essential to obtain high inhibition of pathogens either against fungi or bacteria [32,38,39].

Patulin, the mycotoxin produced by *P. expansum,* is very toxic to humans and has high prevalence in apple-based products. This mycotoxin is essential to the growth of the fungus in apple contributing to its pathogenicity [40]. It is thus important to control the presence of this mycotoxin when controlling the pathogen growth. Different studies have shown that some yeasts such as *Rhodotorula mucilaginosa*, *Pichia caribbica*, *Rhodosporium paludigenum* were able to reduce the content of this mycotoxin [24,41,42]. However, very few studies have been able to find strains capable to inhibit the growth of *P. expansum* and also reduce the mycotoxin content directly, not only by restricting the fungus specific production. For this purpose, tests were performed with the three selected strains in liquid culture medium having a high concentration of patulin (1000 µg/mL). Although yeasts could grow in the presence of a high concentration of patulin, it slowed down the growth rate of yeast and reduced the final concentration of viable yeasts between 0.5 and 1 log. This finding is expected since patulin is a secondary metabolite that *P. expansum* produces to improve its pathogenicity and virulence as a defense mechanism against other organisms [40,43]. Furthermore, these results are in agreement with those found by other authors where the growth of *S. cerevisiae* and *Kodameae ohmeri* was reduced in apple juice and YEPD liquid medium, both with patulin added [13,44]. All the selected yeasts in the present study were capable to reduce patulin content in liquid medium artificially contaminated, Y29 being the most effective with 97% of concentration reduction. In 120 h of incubation, for Y24 and Y29, patulin levels were below the limits established by the EFSA considering the apple juice content regulation of 50 µg/Kg Different studies have elucidated that antagonistic yeasts can reduce patulin from a contaminated medium by different mechanisms. These are: (i) patulin adsorption at the yeast cell wall, (ii) patulin uptake into yeast cells or (iii) patulin biotransformation into less toxic metabolites [45,46]. The mechanism used therefore depends on the BCA used [41].

In this case, patulin was not detected in the intracellular metabolites nor in the yeast cell wall on any analyze, indicating that the mycotoxin was not absorbed and that presumably was subjected to a biological degradation process. This is in accordance with the results of other authors that also studied the ability of different *Metschnikowia* spp. strains such as *M. pulcherrima* or *M. fructicola* in patulin detoxification [32,47]. Other studies with different yeasts have shown that the main products of patulin biodegradation are less toxic compounds than patulin itself. The metabolism of patulin to desoxypatulinic, E-ascladiol or Z-ascladiol by yeasts has been previously reported [25,44]. Some of these compounds are to be expected from the biodegradation of the three strains selected in this work, although further studies are needed to confirm that hypothesis.

To sum up, isolated yeasts can be effective in reducing patulin accumulation in apples through their indirect effect on the reduction of *P. expansum* growth and their direct effect in the patulin biodegradation. Among yeasts, Y29 showed the best performance in both effects. Therefore, Y29 was selected to be applied as a BCA on apples inoculated with *P. expansum* and stored for 17 days at 21 °C. Y29 was able to delay the disease occasioned by *P. expansum* in apples. There are a couple of aspects than can contribute to the success of Y29 as BCA on apples. The nutritional environment of the apple wound could be favorable to the development of *M. pulcherrima* and thus, it could colonize fruit tissues rapidly competing with pathogens. In this regard, Spadaro et al. (2013) [32] concluded that the antifungal action of *M. pulcherrima* strain on “Golden Delicious” apples was better than on other apple cultivars. They attributed this to the high soluble solid content of Golden Delicious apples, which is related to a higher efficacy of BCA. These authors pointed out that one of the main mechanisms of action of these yeasts is competition for nutrients (not only for iron ions) and specially for carbon sources, such as sugars [32]. Moreover, the efficacy of the selected yeast reducing pathogen growth on apples could also be related to yeast were isolated from apple skin, the apple skin, an environment in which yeasts were already able to grow as other authors have previously reported [10]. However, during the last few days of storage, pathogen reduction decreased (Figure 6a). This could be due to high storage temperatures resulted in a low efficacy of the BCA. In fact, fungi stored close to its optimal temperature can increase its growth rate resulting in higher aggressiveness [48,49]. Experiments at low temperatures closed to those applied in industrial warehouses will require longer storage period in order to demonstrate the BCA effectiveness. Future prospects involve to increase the antifungal effectiveness of Y29 in apples contaminated with *P. expansum* by increasing the viability of yeasts or their adhesive properties on apple skin.

Other authors have studied the kinetic of the patulin production in Golden delicious apples artificially inoculated with *P. expansum* during storage [50]. Patulin was not detected for the first three days in apples and its content increased correlated with the developmental profiles of the blue mold observed on apples as occurs in the present work. Indeed, differences in biochemical characteristics of apples, such as sugar concentration, phenolic compounds and pH can affect the growth and production of secondary metabolites by this fungal species [51]. Different studies in Golden delicious apples pointed out that patulin is a virulence factor that helps *P. expansum* to grow [43,50]. On the other hand, the ability of yeast strains to biodegrade patulin has been demonstrated in Section 2.4. The Y29 strain is able to reduce patulin content when applied on apple inoculated with *P. expansum*. However, it is not possible to know which proportion of the lower amount of patulin observed in apples with BCA corresponds to a biodegradation or a non-production of this patulin by *P. expansum*. Further studies are needed to determine and quantify the biodegradation products of patulin in order to draw conclusive results on this reduction.

## 4. Conclusions

The development of naturally efficient control tools that reduce the incidence of *P. expansum* remains essential to avoid food loss of harvested fruits. The results of this study provide evidence of the high potential of *M. pulcherrima* isolated strains from apples as biocontrol agent that not only reduced *P. expansum* incidence but also have the ability to biodegrade patulin into less toxic metabolites. The biocontrol efficacy of the selected yeast in reducing the growth of *P. expansum*, and in reducing patulin content under in vitro conditions have been demonstrated. Y29 exhibited the best results in both in vitro studies. Therefore, it was chosen for applying on stored apple and showed a low but significant inhibition of blue mold decay on wounded inoculated apples by comparison to control. These results are encouraging since the degree of fruit damage and pathogen inoculum level used in this work were far worse than those normally occurring in apples. The isolated strains could be applied as a hurdle with the purpose of inhibiting growth of *P. expansum* and its mycotoxin content on fruits. Studies comprising the incorporation of the BCA into edible coating formulations to improve and maintain the concentration of viable yeasts on the fruit surface are being carried out.

## 5. Materials and Methods

### 5.1. Isolation and Qualitative Confrontation Test

Yeasts used in this study, were isolated from the skin of apples of the variety “Bedan”, in an orchard situated in Brittany, France [27]. A screening was done with 90 isolated yeasts to evaluate their antifungal potential against *Penicillium expansum*. To study the antifungal activity, a qualitative confrontation test was performed. Yeast strains were grown in 10 mL of yeast extract and glucose liquid medium (YEG) (9% yeast extract (Biokar diagnostics, Allonne, France) and 20% glucose (D(+) glucose monohydrate, Merk, Darmstadt, Germany)) at 30 °C for 24 h.

Patulin producing-*Penicillium expansum* (NRRL 35695) was grown on potato dextrose agar (PDA) (Biokar diagnostics, France) in Petri dishes for 7 days at 28 °C. The inoculum was collected by flooding the surface of the plates with sterile peptone water with Tween 80 (0.05% *v*/*v*) (Sigma, Lezennes, France) and then scraping the surface with a spatula. A 5 mL sample of the mold culture suspension was filtered through sterile cotton in order to remove mycelium and transferred to a sterile tube and shaken to obtain a homogeneous suspension. Several dilutions were made to obtain the targeted spore concentration (10^5^ spores/mL) determined using a Thoma cell counting chamber. Then, an amount of 100 µL *Penicillium expansum* suspension (10^5^ spores/mL) were spread on surface of PDA plates. Then, wells were hollowed in the agar using a Durham and 30 µL of each yeast isolates cultured overnight were deposited in the wells l s. A negative control was done using 30 µL of YEG medium. Plates were incubated for 7 days at 25 °C. Three parallel repetitions were performed evaluating two wells per Petri dish. Yeasts which exhibited an inhibition halo around wells were selected for the following studies.

All microbiological products were provided by Sigma (Lezennes, France).

### 5.2. Molecular Analysis

Yeast total genomic DNA was obtained according to standard procedures [52].

#### 5.2.1. Identification

##### Amplification Reactions

PCR amplification of the ITS1-5.8S-ITS2 region of rDNA with the universal primers ITS1 (5′-TCCTCCGCTTATTGATATGC-3′) and ITS4 (5′-TCCGTAGGTGAACCTGCGG-3′) were carried out according to Kurtzman et al. (1998) and Esteve-Zarzoso et al. (1999) [53,54].

##### Sequence Analysis of the ITS1-5.8S-ITS2 Region

PCR amplification of the ITS1-5.8S-ITS2 region were sequenced. PCR products were purified using the NucleoFast Kit (Macherey-Nagel, Düren, Germany) following the manufacturer’s instructions, and were subsequently sequenced through Eurofino Genomics Mix 2seg Sanger sequencing service according to Pérez-Través et al. (2014) [55]. The corresponding sequences, both 5′-3′and 3′-5′reads, were aligned and analyzed with the MEGA 5.0 software. The identification of the closest relatives was obtained from Gen Bank database using NCBI BLAST searching tool. The sequences were deposited in the GenBank database with assigned accession numbers.

#### 5.2.2. Characterization by PCR Fingerprinting Assay

Mitochondrial DNA-restriction fragment length polymorphism (mt DNA-RFLP) analysis was performed by the method of Querol et al. (1992) [52]. Two microliters of DNA were digested with the restriction endonucleases that recognize 5 bp (Hinfl) (Thermo scientific, Vilnius, Lithuania) according to the instructions of the supplier. This enzyme recognizes a large number of sites in the yeast nuclear DNA but few sites in the mt DNA. Restriction fragments were separated in 0.8% agarose gel electrophoresis and visualized in a UV transilluminator after staining.

### 5.3. Antifungal Activity of Yeasts

The minimum initial inoculum of selected yeasts (called Y33, Y29 and Y24) able to reduce microbial growth of *Penicillium expansum* was determined in PDA plates. Yeast strains were precultured in 10 mL of YEG liquid medium at 28 °C overnight. Yeast cells were collected by centrifugation at 8000× *g* for 10 min at 4 °C and washed twice with physiological water. Then, the pellet was suspended on 1 mL physiological water (obtaining a final concentration of 10^8^ CFU/mL verified by plate count) and five serial decimal dilutions were made with each strain. 100 µL of the preculture and of each dilution were spread on the surface of PDA plates. It must be pointed out that the experiment was carried out in petri dishes with a diameter of 9 cm (total area 63.6 cm^2^) and yeast concentration was expressed as CFU/plate. Prior to the experiment, *P. expansum* was precultured for 7 days at 25 °C in PDA. An amount of 20 µL *P. expansum* conidial suspension (10^5^ spores/mL) was spotted in the center of the plates previously inoculated with yeasts. Control plates without yeasts were prepared simultaneously. Plates were incubated at 25 °C for 21 days. Mold diameters were measured periodically during the storage period. Three parallel repetitions were performed.

### 5.4. Biodegradation of Patulin by Antagonistic Yeasts

#### 5.4.1. Microorganisms and Culture Conditions

The patulin biodegradation ability of the previously selected yeasts was tested adapting the protocol of Reddy et al. (2011) [47]. 20 mL of peptone malt extract broth (PM) (1% sucrose (Sigma, Buchs, Switherland), 0.5% yeast extract, 0.5% peptone, and 0.2% malt extract (Biokar diagnostics, Allonne, France) were poured into 100 mL flasks containing patulin standard from 100 µg/mL in acetonitrile (Libios, Vindry-sur-Turdine, France) in order to achieve a final concentration of 1000 µg/L of patulin. A volume of 100 µL of 10^8^ CFU/mL of each yeast strain were inoculated in different flasks and incubated at 25 °C for 120 h at 200 rpm shaking conditions in the light. Controls consisted of noninnoculated broth supplemented with patulin. Cells were collected by centrifugation at 5000× *g* for 10 min at 4 °C at different incubation times, 24, 48, 72 h and 120 h. Cell free supernatant (CFS) was used for measuring patulin reduction. The patulin measured in the cell pellet was used to confirm if an adsorption in the cell wall or an absorption into the cell occurs. Four parallel repetitions were performed in triplicate.

#### 5.4.2. Influence of Patulin on Yeast Cell Viability

The viability of yeast isolates Y24, Y29 and Y33 was determined after 24, 48, 72 and 120 h of incubation at 25 °C in PM medium with patulin (1000 µg·L^−1^) and without patulin (control). Serial dilutions with physiological water were made and plated in Petri dishes previously filled with 15 mL of PDA agar, and they were incubated at 25 °C for 2 days to evaluate survival of yeast cells. The test was performed in four parallel repetitions.

#### 5.4.3. HPLC MS/MS Analysis

HPLC/MS (ACQUITY ^®^ TQD, Waters, Milford, MA, USA) was used to detect and quantify patulin in the samples. The chromatographic separation was achieved on a C_18_ BEH (2.1 × 100 mm, 1.7 μm) (Waters, Milford, MA, USA) column at 30 °C with a flow rate of 0.3 mL/min. The injection was made with a volume of 5 µL. Eluent A was HPLC-grade water and eluent B was acetonitrile (HPLC MS grade, Sigma-Aldrich, St. Louis, MO, USA).

Elution conditions were as follows: a 1 min isocratic passage of solvent B from 100 to 2%, a 3 min gradient increase of solvent B from 2 to 90%, 1 min isocratic passage of solvent B at 90%, a 1 min gradient decrease of solvent B from 90 to 2 and re-equilibration at 2% solvent B for 4 min.

Electrospray ionization was the ionization source. The parameters were as follows: Source temperature: 120 °C, desolvation temperature: 300 °C, Cone gas: 60 L/h, desolvatation gas flow: 800 L/h. Spectra were acquired in negative ionization selected reaction monitoring (SIR) mode with interchannel delay of 0.050 s.

#### 5.4.4. Patulin Cell Adsorption or Absorption

A freezing-thawing in methanol procedure, adapted from Canelas et al. (2009), was used to extract intracellular metabolites (IM) from yeast cells [56]. Pellets obtained from experiment described in Section 5.4.1. were resuspended in 2.5 mL of 100% methanol (Honeywell, Offenbach, Germany) precooled to −80 °C; then, the solution was frozen for 3–5 min in liquid nitrogen and thawed on ice for 3–5 min. Three freeze-thaw cycles were done before centrifuging at 5000× *g* for 5 min at −9 °C. The supernatants were then collected separately, and once more, 2.5 mL of methanol were added to the pellet to extract remaining patulin. Samples were vortexed for 30 s and centrifuged. The supernatants were pooled with the supernatants from the round of extraction. The combined extracts were evaporated using a nitrogen flux and dissolved in 2 mL of water with 0.1% of acetic acid and filtered. The patulin concentration of the methanolic extracts of the intracellular metabolites was determined by HPLC/MS as described as described in Section 5.4.3. The remaining pellet consisting of cell walls was sonicated with physiological water for 20 min. The cell wall suspension (CWS) was then filtered and patulin concentration was determined by HPLC/MS as described above.

### 5.5. Antifungal Activity of the Strain Y29 Applied on Apples

#### 5.5.1. Strain Y29 Application and Antifungal Activity

Golden delicious apples were purchased from a local supermarket and used within 24 h. Fruits without injuries and with similar maturity (in terms of color and firmness) and size (200 g ± 20) were selected. Then apples were immersed in 2% sodium hypochlorite solution for 2 min, then rinsed in distilled water and dried at room temperature to disinfect the surface. Finally, four wounds (3 mm diameter × 3 mm deep) were artificially made along the equator of each apple with a sterile needle. Then, a suspension of Y29 at a concentration of 10^8^ CFU·mL^−1^ in sterile water was prepared following the methodology described in Section 5.3. Apples were dipped in Y29 suspension for 2 min and dried under laminar flow for 2 h. Wounded apples dipped in water without BCA were used as control. Once dried, wounds were inoculated with 20 µL of *P. expansum* at 10^5^ spores/mL. Apples dipped in Y29 suspension and in water were also prepared without *P. expansum* in order to observe if potential changes occur on wounds due to oxidative reactions or yeasts colonization. Apples were stored at 21 °C for 17 days. The disease incidence was then expressed as the measure of *P. expansum* invasion diameter. The test was performed in four parallel repetitions with four wounds per apple.

#### 5.5.2. Yeasts Viability after Drying

Yeast survival in apple surface after drying was studied. A sterile knife was used to remove wounds from each apple and were placed with the whole apple in a sterile plastic bag with 100 mL of sterile distilled water. Apples were then rinsed inside the bag, rubbing them with the hands for 3 min. Then, apples were aseptically removed from bags. The “apple washing water” with the wounds were stomached for 2 min in order to extract yeasts from the wounds. Serial dilutions were made and plated in yeast extract, glucose and chloramphenicol agar (YEG-C). Colonies were counted after incubation for 48 h at 30 °C.

### 5.6. Patulin Quantification on Apple

After 17 days of storage, apples from the previous test were used to quantify patulin. Methodology was adapted from Tannous et al., 2015. Apples were placed individually in sterile plastic bag and were mashed to homogenize in a stomacher. Five g of apple puree were weighed and were mixed with 5 mL of distilled water, and 75 µL of pectinase (Pectinase from Aspergillus aculeatus, Sigma, Søborg, Denmark) on a falcon tube. The mixture was homogenized and let overnight at room temperature. After that, samples were centrifuged at 4500× *g* for 5 min and 5 mL of the supernatant were recovered for make the extraction. The patulin was then extracted and prepared for high-performance liquid chromatography (HPLC) following the next steps.

#### 5.6.1. Patulin Extraction

About 5 mL of the previous supernatant were transferred separately into Falcon flask of 50 mL containing 2 g of sodium bicarbonate (Fluka, St. Quentin Fallavier, France) and 15 g sodium sulphate anhydrous (VWR Chemicals Fontenay-Sous-Bois, France). Fifteen milliliters of a mixture of ethylacetate (VWR chemicals, Fontenay-Sous-Bois, France) and hexane (Fisher scientific, Loughborough, UK) in proportion (60/40) were added before shaking for 10 min. Then, samples were centrifuged at 3000× *g* for 10 min. The organic layer was recovered and completely evaporated under nitrogen stream. The dry extract was suspended in 9 mL of 50% acetic acid solution and sonicated for 30 min. Finally, 1 mL of an internal standard was added, and samples were filtered through a 0.45 mm syringe filter into a clean 2 mL vial.

#### 5.6.2. HPLC MS/MS Analysis

HPLC/MS-MS (LCMS 8040 Shimadzu) was used to detect and quantify patulin in the samples. The data were analyzed using Labsolution software (Shimadzu, Kyoto, Japan). The chromatographic separation was carried out on Kinetex 2.6 μm C18 100A 50 × 2.1 mm ID column (Phenomenex, Hong Kong, China). Column temperature was maintained at 50 °C and injection was made with a volume of 50 μL. The mobile phase A consisted of 90% water 10% mobile phase B consisted acetonitrile (HPLC MS grade, Sigma, St. Quentin Fallavier, France).

Electrospray ionization was the ionization source. The parameters were as follows: positive and negative ionization ES+ and ES-, MRM (Multiple Reaction Monitoring) mode, Desolvation line: 250 °C, Heater block: 400 °C NEB gas: 3 L/min, Drying gas: 15 L/min.

### 5.7. Statistical Analysis

One-way analyses of variance were carried out. The SPSS computer program (SPSS Inc., Chicago, IL, USA) was used. Differences in pairs of mean values were evaluated by the Tukey b test for a confidence interval of 95%. In the case of Figure 6a, the data were analyzed by F-test to test the equality of two variances, followed by Student’s *t*-test to evaluate the differences of the mean between sample and control for a confidence interval of 95%. Data were represented as the average ± standard deviation.

## Data Availability

The data presented in this study are available on request from the corresponding author.

## Supplementary Materials

The following are available online at https://www.mdpi.com/article/10.3390/toxins13060397/s1, Figure S1: PCR fragment of 5.8S genes (ITS region) of isolated yeasts. Lanes m correspond to 100 bp DNA ladder.
