# Supplementary material for: New Isolated Metschnikowia pulcherrima Strains from Apples for Postharvest Biocontrol of Penicillium expansum and Patulin Accumulation
Source: Toxins (Basel). 2021 Jun 2;13(6):397. doi: 10.3390/toxins13060397 (PMC8229137; doi:10.3390/toxins13060397)
Supplement: Supplementary file 1 [file toxins-13-00397-s001.zip › toxins-1217252-SI.pdf]

Supplementary Materials: New Isolated *Metschnikowia pulcherrima* Strains from Apples for Postharvest Biocontrol of *Penicillium expansum* and Patulin Accumulation

Laura Settier-Ramírez, Gracia López-Carballo, Pilar Hernández-Muñoz, Angélique Fontana, Caro-line Strub and Sabine Schorr-Galindo

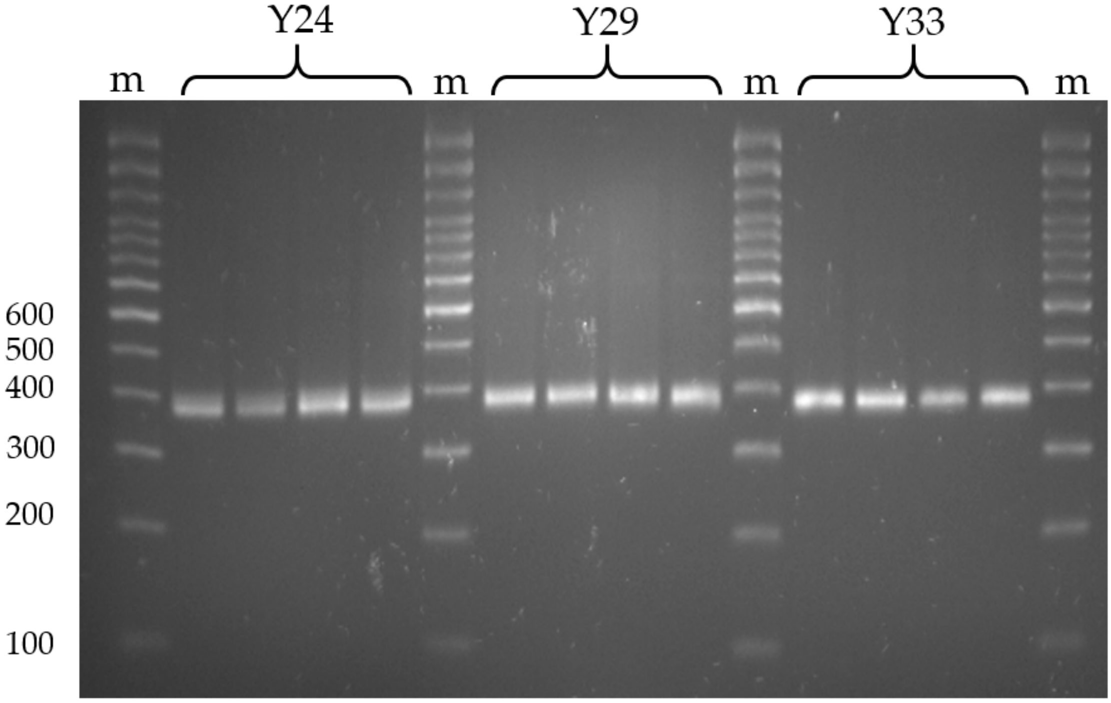

Figure S1. PCR fragment of 5.8S genes (ITS region) of isolated yeasts. Lanes m correspond to 100 bp DNA ladder.
